# Aqueous Extract from *Hibiscus sabdariffa* Linnaeus Ameliorate Diabetic Nephropathy via Regulating Oxidative Status and Akt/Bad/14-3-3**γ** in an Experimental Animal Model

**DOI:** 10.1093/ecam/nep181

**Published:** 2011-02-20

**Authors:** Shou-Chieh Wang, Shiow-Fen Lee, Chau-Jong Wang, Chao-Hsin Lee, Wen-Chin Lee, Huei-Jane Lee

**Affiliations:** ^1^Institute of Medicine, Medical College, Chung Shan Medical University, Taiwan; ^2^Division of Nephrology, Department of Internal Medicine, Kuang Tien General Hospital, Taiwan; ^3^Institute of Biomedical Nutrition, College of Medicine and Nursing, Hung Kuang University, Taichung County, Taiwan; ^4^Department of Pediatrics, Taichung Veterans General Hospital, Taiwan; ^5^Department of Biochemistry, School of Medicine, Medical College, Chung Shan Medical University, Taiwan; ^6^Division of Orthopaedics, Lee's Medical Corporation, Taichung Hsien, Taiwan; ^7^Division of Nephrology, Department of Internal Medicine, Show-Chwan Memorial Hospital, Changhua, Taiwan; ^8^Department of Life Sciences, National Chung Hsing University, Taiwan; ^9^Clinical laboratory, Chung Shan Medical University Hospital, Taiwan

## Abstract

Several studies point out that oxidative stress maybe a major culprit in diabetic nephropathy. Aqueous extract of *Hibiscus sabdariffa* L. (HSE) has been demonstrated as having beneficial effects on anti-oxidation and lipid-lowering in experimental studies. This study aimed at investigating the effects of *Hibiscus sabdariffa* L. on diabetic nephropathy in streptozotocin induced type 1 diabetic rats. Our results show that HSE is capable of reducing lipid peroxidation, increasing catalase and glutathione activities significantly in diabetic kidney, and decreasing the plasma levels of triglyceride, low-density lipoprotein (LDL) and increasing high-density lipoprotein (HDL) value. In histological examination, HSE improves hyperglycemia-caused osmotic diuresis in renal proximal convoluted tubules (defined as hydropic change) in diabetic rats. The study also reveals that up-regulation of Akt/Bad/14-3-3**γ** and NF-**κ**B-mediated transcription might be involved. In conclusion, our results show that HSE possesses the potential effects to ameliorate diabetic nephropathy via improving oxidative status and regulating Akt/Bad/14-3-3**γ** signaling.

## 1. Introduction

Diabetic mellitus (DM) is a consequence of chronic metabolic aberrations including hyperlipidemia. High glucose facilitating the glycolysis and adenosine triphosphate generation would cause huge reactive oxygen species (ROS) production [1]. Under physiological circumstances, ROS involve some signaling molecules and follow defense mechanisms such as phagocytosis, neutrophil function and shear-stress induced vasorelaxation. However, excessive oxidative stress could damage proteins, lipids, and DNA and eliminate anti-oxidative enzymes or molecules [2]. Ujihara et al. observed that oxidized low-density lipoprotein (LDL) level was significantly higher in diabetic patients with macroalbuminuria. They suggested that oxidized-LDL might play an important role in diabetic nephropathy [3]. Experimentally, the oxidative level of LDL can be determined by detecting lipid peroxidation. Antioxidant defense mechanisms include free radical scavengers and enzyme systems, such as superoxide dismutase (SOD), glutathione peroxidase (GSH) and catalase (CAT). Previous studies showed that these anti-oxidative molecules were reduced in diabetes.

In addition to the devastating damage effect on macromolecules, oxidative stress can be involved in cellular signal transduction including Akt signaling pathway [4, 5]. Akt is a principal mediator of biological functions of insulin in glucose metabolism. Phosphorylated Akt can regulate apoptosis via activating Bad to associate with 14-3-3*γ* protein and also to activate nuclear factor-kappa B (NF-*κ*B) to regulate transcription [6–9]. Recent studies showed the importance of phosphoinositide 3-kinases (PI3-K) and Akt signaling pathway in diabetic nephropathy including regulation of renal mesangial hypertrophy and renal tubular cells proteolysis [10, 11]. It has been well known that hyperglycemia and insulin could modulate Akt activity in diabetic renal tissue [12–14]. However, the results are not compatible. Furthermore, the correlation between oxidative stress and Akt signaling in diabetic renal tissues has not been well clarified.

A wide variety of natural products have been found to possess capacity to control metabolic problems and oxidative stress in diabetes. *Hibiscus sabdariffa* Linnaeus (*Malvaceae*, local name Roselle) is usually used as a beverage in Southeast Asia. The constituents in the flowers of Hibiscus species are polyphenolic acids, flavonoids, and anthocyanins. Previous studies found that HSE possessed anti-oxidative characteristics [15] and had anti-atherosclerotic effects [16]. Recent pharmacological studies also showed that HSE significantly reduced blood pressure in humans [17] and in experimental animals [18].

The aim of this study is to assess the effect of HSE on anti-oxidation and on regulation of Akt signaling pathway in diabetic nephropathy. The morphologic change of renal tissues is observed. The GSH level, CAT activity and lipid peroxidation are measured in renal tissues. The Akt/Bad/14-3-3*γ* signaling cascade and NF-*κ*B are also examined.

## 2. Methods

### 2.1. Preparation of HSE

*Hibiscus sabdariffa* L. was purchased from the Taitung Hsien Farmers' Association and identified by Associate Professor Yi-Ching Li, Chung Shan Medical University. A voucher specimen has been kept for future reference at the Department of Pharmacology, Chung Shan Medical University. HSE was prepared as previously described [16]. Briefly, 150 g of dried flowers was macerated with hot water (95°C, 6000 mL) for 2 hours and the aqueous extract was evaporated under vacuum at −85°C. The extracted solution was filtered and then lyophilized to obtain 75 g of HSE and stored at 4°C before use. The concentration of total phenols was analyzed according to the Folin-Ciocalteau method [19]. Briefly, HSE (0.1 mg) was dissolved in 1 mL of distilled water, and then mixed with Folin–Ciocalteu reagent (2 N, 0.5 mL) thoroughly. After an interval of 3 min, 3 mL of 2% Na_2_CO_3_ solution was added, and the mixture was allowed to stand for 15 min with intermittent mixing. The absorbance of the mixture at 750 nm was measured on a Hitachi spectrophotometer (U-3210) with gallic acid as the standard. Total anthocyanins content in HSE was determined using the Fuleki and Francis method [20]. Ten milliliters of HSE (1 mg mL^−1^) was diluted to 50 mL with the pH 1.0 and 4.5 buffers, respectively. The O.D. of the samples was measured at 535 nm, using distilled water as blank. The O.D. difference was obtained by subtracting the total O.D. at pH 4.5 from the total O.D. at pH 1.0. Both values were calculated from the O.D. readings using the appropriate dilution and calculation factors. Total flavonoids content was determined by the Jia method [21] using rutin as a standard. Half of a milliliter of the HSE (1 mg mL^−1^) was diluted with 1.25 mL of distilled water. Then, 75 *μ*L of a 5% NaNO_2_ solution was added to the mixture. After 6 min, 150 *μ*L of a 10% AlCl_3_ *·*6H_2_O solution was added, and the mixture was allowed to stand for another 5 min. A 0.5 mL amount of 1 M NaOH was added, and the total was made up to 2.5 mL with distilled water. The solution was well mixed, and the absorbance was measured immediately against the prepared blank at 510 nm. The final extract (HSE) was composed of 1.43% flavonoids, 2.5% anthocyanins, and 1.7% polyphenolic acid, as measured by quantitative analysis.

### 2.2. Animal Treatment and Experimental Design

All animal experimental protocol used in this study was approved by the Institutional Animal Care and Use Committee of the Chung Shan Medical University (IACUC, CSMC), Taichung, Taiwan. Male Sprague-Dawley (SD) rats (180 ± 10 g) used in the studies was purchased from the National Laboratory Animal Breeding and Research Center (Taipei, Taiwan). The animals were housed in laboratory conditions (18–23°C, humidity 55–60%, 12 hours light/dark cycle) for at least 1 week before each study. Rats were made diabetic by intraperitoneal injection of STZ (dissolved in 0.05 M of citrate buffer, pH 4.5) one time, 65 mg kg^−1^ body weight. Seventy-two hours after STZ administration, fasting blood was collected from the tail vein of all the animals in the experiment to determine the glucose concentration using a reflectance meter (ACCU-CHEK 503363). Animals which were administered with STZ and those in which glucose concentration was <250 mg dL^−1^ were excluded from the study. Blood glucose and body weight were checked every week. The rats were provided with standardized food (Purina Laboratory Chow, obtained from Purina Mills, Inc., USA) and water ad libitum. The rats were divided into five groups with five rats per group. The control group was fed with a standardized diet without any treatment. Buffer control group, intraperitoneally injected with 0.05 M citrate buffer (pH 4.5) was fed with a standardized diet. STZ group was diabetic rats fed with standardized diet. HSE 100 mg kg^−1^ group was diabetic rats tube-fed with HSE 100 mg kg^−1^ per day. HSE 400 mg kg^−1^ group was diabetic rats tube-fed with HSE 400 mg kg^−1^ per day. After treating with HSE for 8 weeks, the rats were sacrificed. Blood was collected for analysis of blood glucose, plasma lipid profiles and renal function. The kidneys were quickly removed on ice and homogenized in lysis buffer. Aliquotes were stored in −70°C. Total protein concentration in each sample was determined by Coomassie blue assay (KENLOR Industries Inc., CA, USA).

### 2.3. Determination of Glucose, Creatinine (Cr), Albumin and Blood Urea Nitrogen (BUN)

Plasma glucose, creatinine, albumin and Blood Urea Nitrogen (BUN) were measured by enzymatic colorimetric methods using automatic analyzer (Olympus AU2700, Olympus Co., Tokyo, Japan).

### 2.4. Plasma Lipid Measurements

Concentration of total cholesterol, triglyceride (TG), low density lipoprotein-cholesterol (LDL-C) and high density lipoprotein-cholesterol (HDL-C) were measured by enzymatic colorimetric methods using commercial kits (Boeheringer Mannheim, Germany).

### 2.5. Pathological Histology of Kidney

After removal from the rats, renal tissues were immediately fixed in 10% buffered formaldehyde and processed for histological examination by conventional methods with hematoxylin and eosin (H&E) stain. The kidney lesions were observed according to morphology changes, such as hydropic changes seen as pale and swollen. The severity of kidney damage was evaluated by examining the sections under five randomly selected high power fields (×200). Image Pro Plus 4.0 was used to quantify the percentage areas of lesions in kidney specimens.

### 2.6. Thiobarituric Acid-Reacting Substances (TBARS)

Rat lipid peroxidation was determined by measuring the Thiobarituric Acid-Reacting Substances (TBARS). Kidney tissue of 0.5 g of was homogenized with 5 mL of RIPA buffer (1% NP-40, 50 mM Tris-base, 0.1% SDS, 0.5% deoxycholic acid, and 150 mM NaCl, pH 7.5 with 10 *μ*g mL^−1^ leupeptin, 10 *μ*g mL^−1^ PMSF and 17 *μ*g mL^−1^ sodium orthovanadate) and centrifuged (1000 g) for 30 min to obtain supernatant homogenate. Protein content of the supernatant was determined with Bio-Rad protein assay reagent using bovine serum albumin as a standard. The 0.3 mL of homogenate was then added with 0.3 mL of TBA (1% thiobarbituric acid in 0.3% of NaOH) and the mixture was allowed to react for 40 min at 95°C in the dark. After the reaction, samples were analyzed in a Hitachi F2000 spectrophotofluorimeter with excitation at 532 nm and emission at 600 nm. The concentrations of TBARS were expressed as equivalents of malondialdehyde (MDA) represented as *μ*M mg^−1^ protein.

### 2.7. Determination of GSH Content

The kidney GSH content was determined according to the method of Hissin and Hilf [22]. The stock solution of the fluorescent probe *o*-phthalaldehyde (OPT) was freshly prepared in methanol (1 mg mL^−1^). A total of 10 *μ*L of the homogenate was mixed with OPT and incubated in the dark for 15 min. We then monitored the fluorescence intensity with excitation wavelength at 350 nm and emission wavelength at 420 nm. We established a GSH calibration curve to normalize the GSH concentration. The GSH result was expressed as ng mg^−1^ protein.

### 2.8. Catalase Assay

Catalase activity in rat kidney homogenate was assayed according to a previous method [23]. Briefly, 20 *μ*L of homogenate was added to 980 *μ*L of H_2_O_2_ solution (containing 30 *μ*L of ddH_2_0, 50 *μ*L of Tris–HCl-EDTA, pH 8.0 and 900 *μ*L of 10 mM H_2_O_2_). After 10 s at room temperature, the optical density of H_2_O_2_ was recorded at 240 nm for 1 min using a spectrophotometer. A unit of catalase activity was defined in H_2_O_2_ consumed units/mg protein.

### 2.9. Preparation of Cell Extracts and Immunoblot Analysis

Total proteins of tissue samples were extracted by incubating the cells with 200 *μ*L of cold lysis buffer (50 mM Tris–HCl, pH 7.4; 1 mM NaF; 150 mM NaCl; 1 mM EGTA; 2 mM phenylmethylsulfonyl fluoride; 0.1% Triton X-100; and 10 *μ*g mL^−1^ leupeptin) on ice for 30 min, followed by centrifugation at 1000 g for 10 min at 4°C. Protein concentration of the supernatant was measured by Coomassie blue assay. Samples (50 *μ*g of protein) were mixed with 5× sample buffer (0.4 M Tris–HCl, pH 6.8, 0.5 M dithiothreitol, 10% SDS, 50% glycerol and 0.005% bromophenol blue). The mixtures were boiled at 95°C for 5 min and subjected to separation on 8% SDS polyacrylamide gels at a constant voltage of 160 V. Following electrophoresis, proteins on the gel were electro-transferred onto an immobile membrane (nitrocellulose; Millipore Corp., Bedford, MA) with transfer buffer (25 mM Tris–HCl, 192 mM glycine, and 20% methanol). The membrane was blocked with blocking solution (20 mM Tris-base, pH 7.4, 125 mM NaCl, 0.1% Tween 20, and 1% bovine serum albumin). The membranes were then immunoblotted with the primary antibodies (Akt, 1 : 1000; phosphor-Akt, 1 : 1000; Bad, 1 : 1000; phosphor-Bad, 1 : 1000 and 14-3-3*γ*, 1 : 1000, all purchased from Santa Cruz Biotechnology, CA). The reacted bands were revealed by enhanced chemiluminescence using an ECL commercial kit, and the relative photographic negatives by a gel documentation and analysis system (Alpha Imager 2000, Alpha Innotech Corp., San Leandro, CA).

### 2.10. Electrophoretic Mobility Shift Assay (EMSA)

The nuclear protein extracts from kidney tissues were prepared as described previously [24]. Protein concentrations were measured using Coomassie blue assay with bovine serum albumin as standard. Double-stranded oligonucleotides containing the consensus sequence for NF-*κ*B/DNA binding site (Promega, Madison, WI) were end-labeled with chemiluminescence. Nuclear extracts (2 *μ*g) were incubated with Biotin-labeled probe at room temperature for 20 min in a binding buffer containing poly (dI-dC). The specificity of the binding reaction was determined by preincubation either with 100-fold molar excess of unlabeled NF-*κ*B probe or with monoclonal anti-NF-*κ*B antibodies (anti-p65, Santa Cruz Biotechnology). DNA-protein complexes were separated in 6% nondenaturing polyacrylamide gel at 90 V for 2-3 h. After transferring and hybridizing with HRP-conjugated streptavidin, the reacted bands were revealed by enhanced chemiluminescence using an ECL commercial kit, and the relative photographic negatives by a gel documentation and analysis system (Alpha Imager 2000, Alpha Innotech Corp., San Leandro, CA).

### 2.11. Statistical Analysis

Statistical analysis was done using Microsoft Excel 2007 and Sigma plot version 9.0. Results are presented as mean ± SD. Statistical differences were analyzed by Student's *t*-test. *P* < .05 was considered statistically significant.

## 3. Results

### 3.1. Plasma Biochemical Parameters Profiling in Various Rats Groups

The body weight and biochemical markers including lipid profiles were presented in Table 1. There was a remarkable decrease in the body weight of STZ-induced diabetic rats (*P* < .01). The STZ-induced diabetic rats had significantly increased blood glucose concentration compared with rats in control group (*P* < .01). HSE treatment was not able to attenuate the elevated blood glucose value in STZ diabetic rats. There were no differences in plasma sodium (Na), potassium (K) and chloride (Cl) values among control rats, diabetic rats and HSE-treated rats. The diabetic rats had higher values of plasma BUN than control rats. But there was no difference in plasma Cr level between control rats and diabetic rats. HSE treatment had no significant influence on BUN and Cr concentrations. Albumin value declined significantly in STZ diabetic rats. HSE at dose 100 mg kg^−1^ could not improve the albumin value but a higher dose of HSE (400 mg kg^−1^) could restore albumin level remarkably. 

### 3.2. Improvements in Hyperlipidemia by HSE

Among lipid profiles, plasma total cholesterol concentrations (*P* < .01) and TG (*P* < .01) increased prominently in STZ diabetic rats compared to control rats. After HSE treatment, the TG level reduced significantly at HSE dose dependently (100 mg kg^−1^: *P* < .05; 400 mg kg^−1^: *P* < .01). However, HSE treatment did not affect total cholesterol value. Obviously, LDL-C value rose remarkably in STZ diabetic rats (*P* < .01). Although HSE at dose of 100 mg kg^−1^ could not reduce LDL-C level, higher HSE dose at 400 mg kg^−1^ could decrease LDL-C concentration significantly (*P* < .05). In addition, we found HDL-C levels were comparable between control rats and STZ diabetic rats. Only higher dose of HSE (400 mg kg^−1^) could improve HDL-C value considerably (*P* < .05).

### 3.3. Attenuation on Kidney Lesions in STZ-Induced Diabetic Rats by HSE

**Figure 1:**
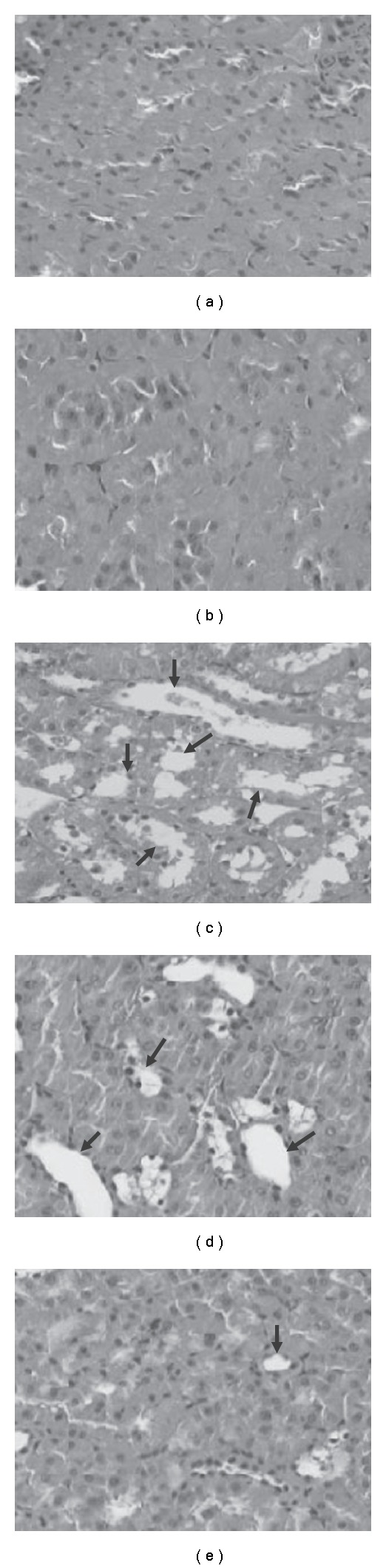
Effects of HSE on diabetic nephropathy in rats. Histological examination (200×) in rat kidney of (a) normal group, (b) citrate buffer group, (c) STZ-treating group, (d) STZ + 100 mg kg^−1^ day^−1^ of HSE, (**E**) STZ + 400 mg kg^−1^ day^−1^ of HSE. Arrows represent the hydropic changes seen as pale and swollen in proximal convoluted tubules.

**Figure 2:**
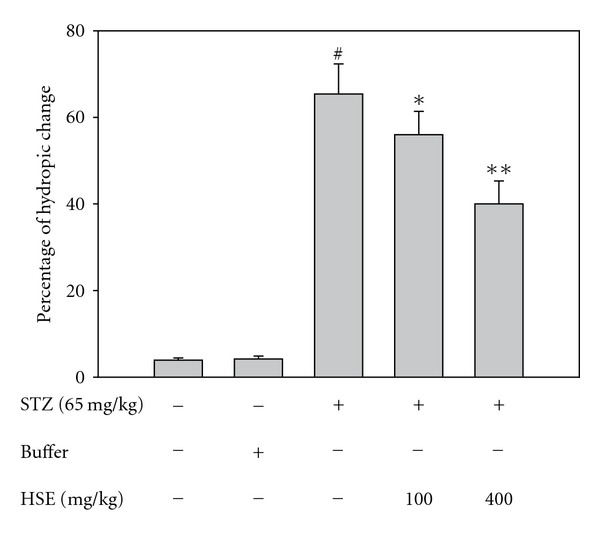
Quantitative determination of the percentage of hydropic change. ^#^ *P* < .05, significant differences compared with the control; *P < .05, **P < .005, compared with the STZ group.

The pathological change of STZ induced diabetic renal tissues was evaluated under light microscope (Figure 1). On histological examination, there was remarkable hydropic change over proximal tubules in renal tissues of STZ induced diabetic rats. Treatment of diabetic rats with HSE 100 and 400 mg kg^−1^, the hydropic change in diabetic renal tissues were decreased prominently (Figure 2). 

### 3.4. HSE Protect Kidney from Oxidative Stress

The levels of MDA, CAT and GSH were depicted in Table 2. MDA extracted from renal tissues of STZ diabetic rats was substantially increased compared to control rats. Treatment of STZ rats with HSE at doses, 100 and 400 mg kg^−1^ significantly diminished MDA formation. In diabetic rats, there was a significant decrease in renal CAT activity (*P* < .01) and GSH level (*P* < .01). HSE at only 100 mg kg^−1^ only significantly improved CAT levels (*P* < .01) in kidney tissues of diabetic rats. But HSE at 400 mg kg^−1^ significantly increased both CAT (*P* < .01) and GSH (*P* < .01) levels. 

### 3.5. Increase in Expressions of Akt/Bad/14-3-3*γ* and NF-*κ*B by HSE

**Figure 3:**
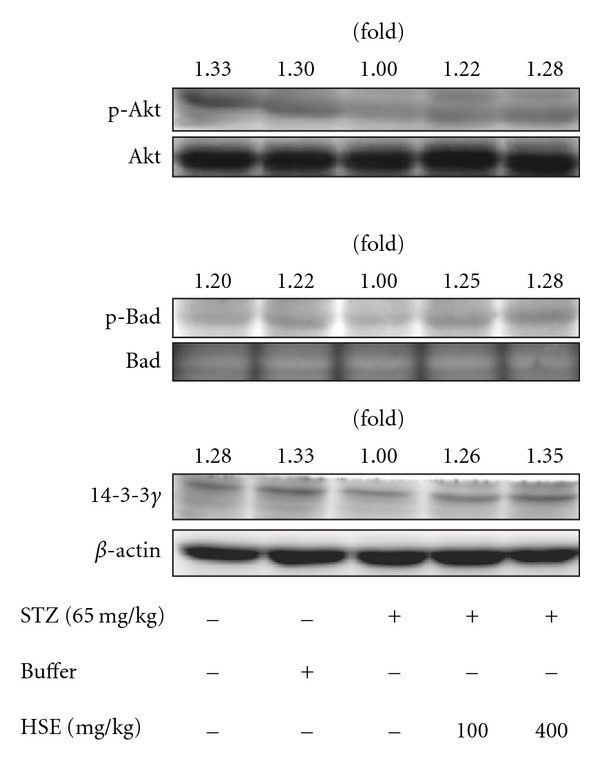
Effect of HSE in the protein expressions of phosphorylated Akt, phosphorylated Bad and 14-3-3*γ* in rat kidneys. The expressions of p-Akt, p-Bad and 14-3-3*γ* were done as described in the text. The data are repeated for three independent examinations.

**Figure 4:**
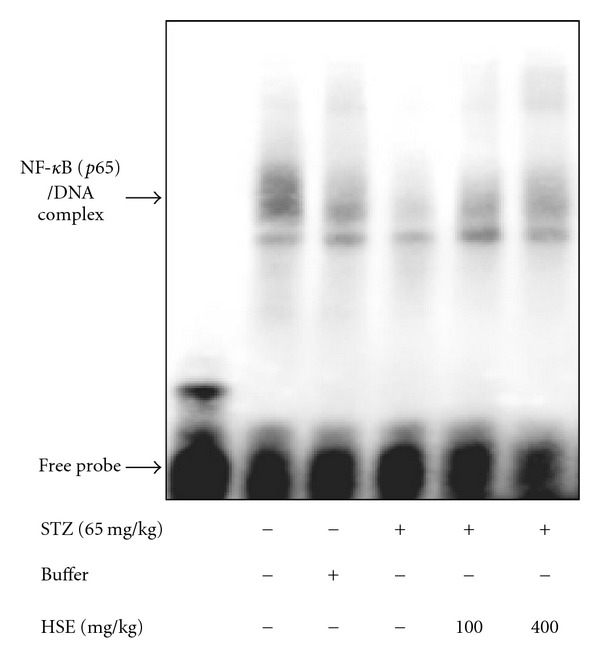
Effect of HSE on the NF-kB activity. The activity of NF-*κ*B was performed with EMSA as described in the text. The data are repeated for three independent examinations.

We measured the expression of Akt signaling cascade. In Figure 3, there was a remarkable decrease by 30% in p-Akt expression in renal tissues of STZ diabetic rats compared to control rats. Both treatments with HSE 100 or 400 mg kg^−1^ increased p-Akt expression by 22% and 28%, respectively. We also found that p-Bad protein expression was significantly declined by 22% in kidney tissues of STZ rats compared to control rats. Similarly, HSE at doses 100 and 400 mg kg^−1^ was found to increase p-Bad protein expression by 25% and 28%, respectively compared to diabetic rats. The expression of 14-3-3*γ* protein in renal tissues of STZ rats was reduced by 33% compared to control rats. We found that treatment with HSE 100 mg kg^−1^ increased the expression of 14-3-3*γ* protein by 26% and HSE 400 mg kg^−1^ by 35% compared to diabetic rats. As shown in Figure 4, although the level of NF-*κ*B activity was decreased in diabetic rats, HSE at doses 100 and 400 mg kg^−1^ increased NF-*κ*B activity compared to diabetic rats, but it was unrelated to AP-1 regulated transcription (data not shown). 

## 4. Discussion

Atherogenic dyslipidemia comprises the triad of high LDL-C, high TG and low HDL-C values and is a part of metabolic syndrome. It is well established that hyperlipidemia is a strong risk factor of cardiovascular disease in type 1 and type 2 diabetic patients [25]. Moreover, hyperlipidemia predicts progressive loss of renal function in chronic kidney disease in both type 1 [26] and type 2 [27] diabetes. In our study, plasma cholesterol, TG and LDL-C levels are increased significantly in STZ diabetic rats, while plasma HDL-C value is found decreased prominently in diabetic rats. This finding is consistent with previous studies of diabetic rats induced by STZ [28] or alloxan [29]. In this study, we found that HSE have the capacity of decreasing TG and LDL-C significantly in STZ diabetic rats. The results are in accordance with previous studies in diabetic [28, 29] or hypercholesterolemic [15, 16] animals. Furthermore, HSE (400 mg kg^−1^) could increase HDL-C level significantly in our experimental animals. It is implied that HSE might possess a promising effect on deceleration of metabolic syndrome in diabetes.

Previous study shows an increasing oxidative stress and reducing anti-oxidative ability in diabetes [30]. Oxidative stress results in glomerular sclerosis, renal tubular injury, proteinuria and leads to gradual loss of renal function [31]. Excessive production of ROS by oxidative stress in hyperglycemic status might result in decline of GSH value [32]. *Hibiscus sabdariffa* L. contained a variety of bioactive compounds with antioxidant properties, such as protocatechuic acid, catechin and (−)-epigallocatechin gallate [33]. They could scavenge harmful free radicals and regenerate other antioxidants to prevent cellular oxidative damages. Protocatechuic acid could attenuate diabetic complications via elevation of GSH and CAT activities in kidney and significant reduction in plasma C-reactive protein, interleukin-6 and other inflammatory cytokines [34]. Previous study also demonstrates that catechin has protective effects on STZ diabetic rats. Catechin treatment significantly reduces albumin excretion rate in diabetic rats and normalizes the interstitial fibrosis completely in diabetic kidney [35]. Previous investigation shows that (−)-epigallocatechin gallate could alleviate renal damage in STZ diabetic rats by suppression of hyperglycemia, proteinuria, and lipid peroxidation [36]. Anthocyanins (including cyanidin and delphinidin) have strong antioxidant activity in a liposomal system [37] and could ameliorate hyperglycemia and insulin sensitivity in diabetic mice [38]. In this study, our data shows HSE could reduce MDA level in kidney tissues significantly, and improve CAT and GSH activities significantly in STZ diabetic rats. The results show that HSE possesses potent antioxidant effects on diabetes.

A detailed pathogenesis of diabetic nephropathy is still not clear. It is considered as primarily a glomerular disease, including thickening of glomerular basement membrane, mesangial expansion and podocyte loss. However, recent evidence demonstrates that chronic hypoxia of the tubulointerstitium has a pathogenic role in diabetic nephropathy [39]. In our study, we did not find typical diabetic pathological change of Kimmelsteil-Wilson nodules in glomerulus. The finding might result from too short duration to induce obvious glomerular changes. Indeed, only minor or even no glomerular change is reported in early diabetic renal tubular injury [40]. In the present study, renal tubules have hydropic change, characterized by pale and swollen change of the proximal convoluted tubules in STZ diabetic rats, as reported previously [41]. The feature maybe a manifestation of osmotic diuresis resulting from high glucose concentration. After HSE administration, significant decrease of hydropic change of renal tubules is noted. Because HSE has no significant effect on blood sugar level in this study, we have good reason for thinking that the antioxidant effect might contribute to attenuation of structural changes in renal tubules.

The capacity of insulin to activate Akt signaling cascade is impaired in hyperglycemia. In type 2 DM, PI3K-Akt pathway is considered a site of insulin resistance [42, 43]. However, in type 1 DM, the Akt activity in a variety of tissues is not yet determined. Sheu et al. demonstrates that hyperglycemia leads to increased phosphorylation and activity of Akt in murine and rat glomerular mesangial cells [14]. In addition, Lee et al. reports that high glucose has sustained stimulation to PI3K activity with following augmented effect of Akt activity in glomerular epithelial cells in STZ diabetic rats [13]. Conversely, some other studies disclose that lack of insulin or hyperglycemia in type 1 DM results in lower Akt activities. Kondo et al. finds decreased Akt protein and phosphorylation of Akt in retinal tissue of STZ diabetic rats [44]. And Laviola et al. also shows decreased phosphorylation of Akt and Akt activity in myocardiocytes of STZ diabetic rats [45]. Moreover, Zdychová et al. reports reduction in phosphorylation and activity of Akt in renal tissues of diabetic animals. The restoration of Akt activity and phosphorylation of Akt is noted after administration of insulin [12]. The conflicting results of Akt expression from previous studies might result from different tissues or individual exposure duration to high glucose in diabetic animals.

In fact, oxidative stress is one of the important factors involved in impairment of Akt activation in hyperglycemia status. Overproduction of H_2_O_2_ in adipocytes would result in significant reduction of Akt phosphorylation in the previous study [46]. Moreover, tumor necrosis factor-*α*, an inflammatory cytokine, has been identified to stimulate natural antagonist of ceramide with suppression of Akt phosphorylation [47]. It has been demonstrated that high glucose-induced oxidative stress by peroxynitrite could inhibit activity of Akt-1 kinase and increase the activity of p38MAP kinase [48]. In our results, expression of p-Akt, p-Bad and 14-3-3*γ* protein is found decreased in STZ diabetic rats compared to normal rats. All of them are found to recover significantly after HSE treatment at dose of 100 or 400 mg kg^−1^. It seems reasonable to suppose that HSE could restore Akt level and following signaling cascade through improvement of oxidative stress in diabetic rats. However, not all previous studies agree that HSE could increase Akt expression. HSE is found to block the PI3-K/Akt pathway with following declined Akt phosphorylation during adipocyte differentiation in 3T3-L1 cells [49]. We have no definite explanations of these disparate results. This issue needs further examination to clarify the relationship between HSE and Akt.

In previous studies, markedly low Akt phosphorylation expression is seen associated with enhancement of apoptosis of high glucose-stressed mesangial cells in STZ diabetic rats [50]. On the other hand, high glucose stimulates increasing p-Akt level and mesangial cells proliferation in diabetic rats [14]. Promotion of cell survival by Akt involves various mechanisms. Akt could directly phosphorylate Bad protein with combination of 14-3-3*γ* protein to activate anti-apoptotic effect [51]. In addition, Akt appears to positively regulate pro-survival transcriptional factors, such as NF-*κ*B, for controlling cell survival [43]. NF-*κ*B could regulate gene expression in inhibition of apoptosis and enhances cell proliferation and angiogenesis [9]. In our study, it might be assumed that increased activity of Akt and NF-*κ*B have an impact on attenuation of diabetic nephropathy through anti-apoptotic effect. Although NF-*κ*B is found to be activated by oxidative stress in diabetic nephropathy [31], and might involve in the pathophysiology of diabetic microangiopathy [52], we speculate that the NF-*κ*B-dependent pathway has different regulations among a variety of tissues during various stages of diabetic nephropathy.

**Figure 5:**
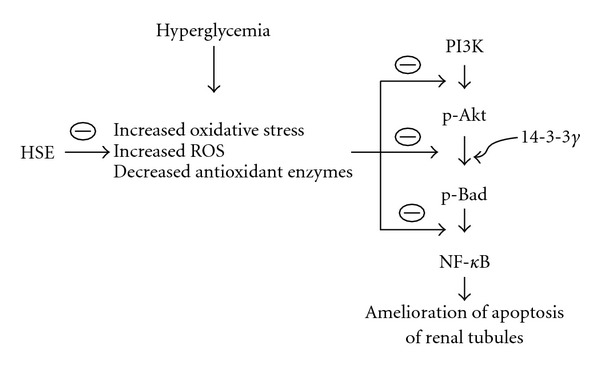
Proposed mechanisms of HSE ameliorating diabetic nephropathy in an experimental animal model. Dashed oval means inhibition.

Taken together, our data show that HSE possesses strong antioxidant properties as well as lipid-lowering effect. HSE not only can significantly decrease TG and LDL-C levels but also increase HDL-C value. Moreover, HSE is demonstrated to have potential attenuation effect on diabetic nephropathy through its anti-oxidative and anti-apoptotic mechanisms (Figure 5). Limited by the relatively small sample size of animals, we need further studies to elucidate the relationship between HSE and Akt pathway in diabetes and to clarify the detailed mechanisms of HSE on improvement in diabetic nephropathy. From our results, HSE shows therapeutic promise in amelioration of hyperlipidemia and prevention of metabolic syndrome in diabetic patients. 

## Funding

National Science Council Grant (NSC96-2321-B-040-003) and the Chung Shan Medical University Grant (CSMU94-OM-B-018 and CSMU93-OM-B-024).

## Figures and Tables

**Table 1:** Physiological and biochemical parameters of rats in various groups (mean ± standard deviation).

	Normal	Control	STZ	STZ + HSE	STZ + HSE
				(100 mg kg^−1^)	(400 mg kg^−1^)
Body weight (g)	393.33 ± 11.55	406.67 ± 20.82	240.00 ± 14.14 ^(b)^	205.00 ± 36.97 ^(b)^	206.00 ± 28.81 ^(b)^
Glucose (mg dL^−1^)	145.33 ± 17.21	141.33 ± 11.06	630.00 ± 77.19 ^(b)^	598.25 ± 67.29 ^(b)^	607.65 ± 16.78 ^(b)^
BUN (mg dL^−1^)	18.20 ± 1.64	15.23 ± 1.33	34.08 ± 5.12 ^(b)^	31.88 ± 6.67 ^(b)^	35.25 ± 3.15 ^(b)^
Creatinine (mg dL^−1^)	0.47 ± 0.06	0.50 ± 0.10	0.52 ± 0.04	0.46 ± 0.05	0.50 ± 0.08
Albumin (g dL^−1^)	3.30 ± 0.10	3.33 ± 0.31	2.84 ± 0.30 ^(b)^	2.90 ± 0.14	3.20 ± 0.08 ^(d)^
Cholesterol (mg dL^−1^)	59.00 ± 5.00	50.00 ± 3.61	106.67 ± 9.87 ^(b)^	101.75 ± 21.93 ^(a)^	108.25 ± 14.93 ^(b)^
Triglyceride (mg dL^−1^)	139.700 ± 65.70	88.00 ± 35.60	859.00 ± 196.00 ^(b)^	363.00 ± 127.80 ^(b),(c)^	265.50 ± 46.90 ^(b),(d)^
HDL-C (mg dL^−1^)	24.00 ± 1.73	22.00 ± 2.65	21.00 ± 5.24	35.80 ± 13.59	54.25 ± 15.13 ^(b),(c)^
LDL-C (mg dL^−1^)	8.67 ± 1.53	9.00 ± 0.00	17.60 ± 3.05 ^(b)^	16.80 ± 6.94	12.50 ± 3.70 ^(c)^
Sodium (mmol l^−1^)	144.30 ± 0.60	148.00 ± 4.00	143.20 ± 1.50	144.80 ± 1.90	144.50 ± 1.30
Potassium (mmol l^−1^)	7.37 ± 0.23	8.27 ± 0.75	6.54 ± 0.58	6.30 ± 0.28	6.10 ± 0.61
Chloride (mmol l^−1^)	101.33 ± 0.58	103.67 ± 2.52	97.00 ± 3.39	98.80 ± 3.49	101.50 ± 1.00

*n* = 5.  ^(a)^ *P* < .05;  ^(b)^ *P* < .01 significantly different from control group;  ^(c)^ *P* < .05,  ^(d)^ *P* < .01 significantly different from STZ group.

**Table 2:** Antioxidant enzyme profiles and marker of oxidative stress in renal tissues of rats from the different study groups (mean ± SD).

	Normal	Buffer	STZ group	STZ + HSE	STZ + HSE
				(100 g kg^−1^)	(400 g kg^−1^)
MDA (M mg^−1^ protein)	2.83 ± 0.78	3.32 ± 0.78	4.04 ± 0.51 ^(a)^	2.74 ± 0.72 ^(d)^	2.31 ± 0.43 ^(d)^
Catalase (Units mg^−1^ protein)	11.21 ± 4.78	11.25 ± 5.22	6.11 ± 3.03 ^(b)^	24.97 ± 6.93 ^(d)^	34.00 ± 8.81 ^(d)^
GSH (ng mg^−1^ protein)	9.28 ± 0.40	9.63 ± 0.33	6.80 ± 0.29 ^(b)^	6.28 ± 0.25	9.25 ± 0.54 ^(d)^

*n* = 5.  ^(a)^ *P* < .05;  ^(b)^ *P* < .01 significant differently from control group;  ^(c)^ *P* < .05;  ^(d)^ *P* < .01 significant differently from STZ group.
